# Platelet-rich gel versus external tissue expansion technique in treating scalp defects: A retrospective study

**DOI:** 10.1097/MD.0000000000036305

**Published:** 2023-12-01

**Authors:** Tao Ai, Jianbai Wang, Yanan Xu

**Affiliations:** a Department of Traumatology, Chongqing Emergency Medical Center/Chongqing University Central Hospital, Chongqing, China.

**Keywords:** external tissue expansion technique, platelet-rich gel, scalp defects, wound repair

## Abstract

Reconstruction of scalp defects is a complicated and challenging procedure for reconstructive surgeons. This retrospective observational study assessed the effectiveness of using platelet-rich gel (PRG) versus the external tissue expansion technique (TET) in reconstructing scalp defects. The clinical data of 24 patients with scalp defects treated with PRG or external TET were collected from September 2018 to March 2022. Data on the wound characteristics, wound healing time, cost of treatment, visual analog scale, and observed wound healing status were collected. The mean wound healing times in the PRG and TET groups were 25.00 ± 5.77 and 13.58 ± 9.68 days, respectively (*P* < .05). The PRG group was significantly more cost-effective than the TET group (*P* < .05). TET treatment significantly increased patients’ postoperative pain, which decreased over time (*P* < .05), while PRG treatment caused no significant change in pain (*P* > .05). The 2 groups showed no tissue depression or color change after wound healing at follow-up, but the hair growth in the TET group was significantly better than that in the PRG group (*P* < .05). Compared with TET treatment of scalp defects, PRG is not only simple and painless but also has a low treatment cost and, more importantly, does not involve the risk of surgery and anesthesia. However, using TET to treat scalp defects requires the careful selection of appropriate cases.

## 1. Introduction

Scalp defects can be caused by several etiologies, such as trauma, burns, pressure ulcers, infections, and tumor resection.^[1]^ Scalp defect wounds with edges >2.5 cm are difficult to suture because the scalp is close to the skull, and there is less subcutaneous soft tissue and poor mobility.^[2]^ Numerous treatment protocols are currently available for scalp defect reconstruction. According to previous studies, small to moderate scalp defects (≤20 cm^2^) can be repaired with local flap transfer, and large ones (>20 cm^2^) can be covered with a free anterolateral femoral flap.^[3,4]^ Additionally, clinical trials have demonstrated the efficacy of artificial dermis,^[5]^ skin-stretching devices,^[6]^ and tissue dilators^[7]^ in treating scalp defects. All the aforementioned methods involve surgery and must be performed under local or general anesthesia. These methods are unsuitable for elderly patients with a poor cardiopulmonary function who cannot tolerate anesthesia. Nonsurgical treatment is the only option for such patients.

As a concentrated platelet product, platelet-rich gel (PRG) is actually platelet-rich plasma (PRP) gel in most literature and is prepared by adding thrombin and calcium gluconate to PRP. It has been widely used in various wound treatments in recent years and has achieved excellent clinical efficacy.^[8,9]^ The mechanism by which PRG promotes wound healing is the same as that of PRP, not only because it can provide a variety of growth factors but also because of its antibacterial effects.^[10]^ The tissue expansion technique (TET) takes advantage of specific skin characteristics, such as extensibility and viscoelasticity, to gradually pull the 2 sides of the wound to the middle and achieve complete closure of the wound within a short period.^[11]^ TET provides a relatively simple and appropriate method for the primary closure of skin defects. TET has gained popularity recently as a surgical technique for treating wounds and has achieved great clinical efficacy, thus becoming an important means for treating various wounds.^[12,13]^

This retrospective observational study was conducted to assess the role of PRG as a nonsurgical treatment for scalp defects combined with skull exposure in comparison with surgical treatment using a skin-stretching device as a control.

## 2. Material and Methods

### 2.1. Study design and setting

Patients with scalp defects treated with PRG and skin-stretching devices at the Chongqing Emergency Center were retrospectively studied between September 2018 and March 2022. Ethical approval for this study was obtained from the ethics committee of the Chongqing Emergency Medical Center.

This study included patients with scalp defects combined with skull exposure caused by trauma, pressure ulcers, and poorly healed incisions. Patients and their families were informed about PRG treatment and surgical operations with skin-stretching devices, and relevant medical documents were signed after obtaining their consent. The inclusion criteria were as follows: scalp defects caused by trauma, pressure ulcers, and poorly healed incisions. The defect wound could not be closed directly because the distance between the wound edge was more than 2.5 cm. Exclusion criteria were exposed titanium mesh wound after cranioplasty, cancerous wound, severe malnutrition (hemoglobin level < 65 g/L, albumin level < 25 g/L), low platelet count, and abnormal coagulation function before treatment.

### 2.2. Autologous PRG preparation and treatment procedures

**Figure 1.**
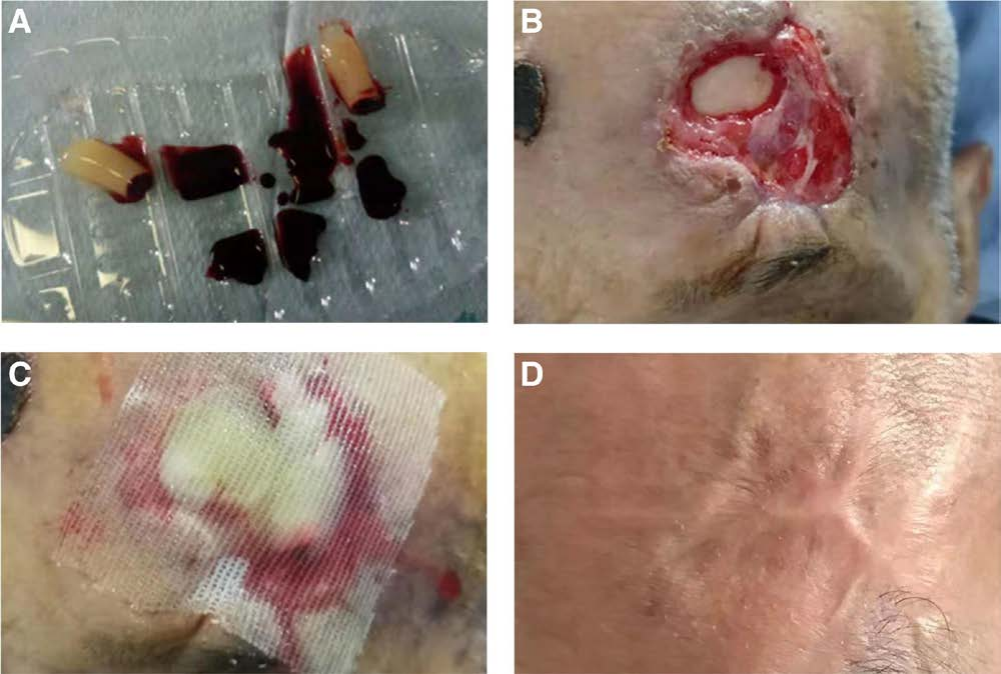
Reconstruction of scalp defect in frontal site using PRG. (A) Preparation of PRG using the one-step centrifugation method. (B) Skull exposed after frontal wound debridement. (C) The wound was filled with PRG and covered with Vaseline gauze. (D) The wound healed completely. PRG = platelet-rich gel.

Centrifuge tubes with a coagulant were used to prepare PRG. Five milliliters of blood was collected in each tube, and the amount of blood required to be collected was determined by the size and depth of the wound. The centrifuge tubes with the collected blood were placed in a centrifuge at 3000 rpm for 5 minutes. After centrifugation, the blood in the tubes was categorized into 3 layers: the upper layer was a light yellow, clear liquid (acellular plasma), the middle layer was a light yellow gel (PRG), and the bottom layer was red blood cells. PRG and red blood cells can be easily separated using tweezers. Before PRG treatment, scalp defects were debrided to remove necrotic tissue and part of the inflammatory granulation tissue. The PRG filled the inside of the wound, and the surface was covered with Vaseline gauze to prevent the liquid containing growth factors from spilling outside the wound. The outermost layer was covered with hydrogel dressing to close the wound, and PRG was replaced every 3 days until the wound healed (Fig. 1).

### 2.3. Surgical procedure and postoperative treatment

**Figure 2.**
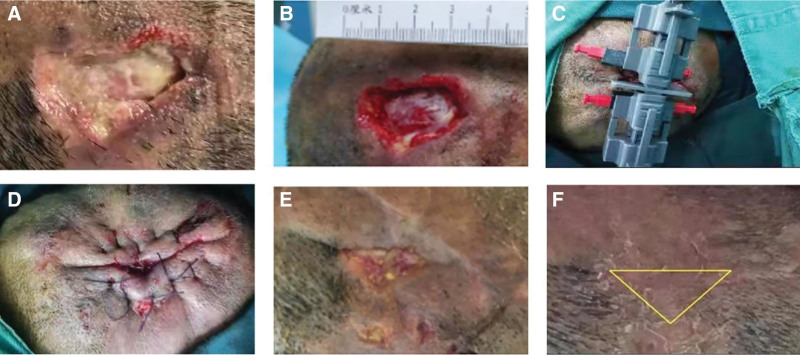
Reconstruction of scalp defect in the occipital site with an external tissue expansion technique. (A) Pressure sore in the occipital site. (B) Scalp defect after debridement. (C) The wound was reconstructed with an external tissue expansion technique. (D) Suture after skin stretching. (E) A small part of the wound remains unhealed after the operation. (F) The wound healed completely after dressing changes.

The operation was performed under local or general anesthesia. First, the wound was debrided to remove necrotic tissue, old granulation tissue, and inflammatory tissue, and the edge of the wound was trimmed. If the pinch test was positive, indicating that the wound could not be closed directly, the scalp defect was closed using an EASApprox skin stretching device (BIOWIM, LTD, Dalian, China). Next, the hook set of the skin stretcher was installed 0.5 cm from the edge of the wound. A force of 0.5 to 3.0 kg was applied to the edge of the incision skin with a skin stretcher for 4 minutes and then relaxed for 1 minute. This procedure can be repeated for several cycles (not >5 times) to reduce the tension of the wound. Finally, the wound was closed by intermittent suture with 2/0 Coated Vicryl Plus Antibacterial Suture (Fig. 2). The dressing was changed every 3 days until the wound healed, and the stitches were removed approximately 7 to 10 days after the operation.

### 2.4. Treatment of the outcome and follow-up

The primary endpoint of the study was the wound healing time. Successful wound healing was defined as the epithelium and fibrous tissues covering the wound without any secretions or granulation formation. The secondary endpoint was assessed using the visual analog scale (VAS) at 6 hours and 3 days postoperatively. The VAS score ranged from 0 to 10, where 0 denotes no pain, 1 to 3 denotes mild pain within tolerable limits, 4 to 6 denotes pain that affects sleep, and 7 to 10 denotes activity and maximum pain that seriously affects appetite and sleep. Another important endpoint was the assessment of the cost-effectiveness of both treatment modalities. The final evaluation of indicators was the wound healing status at 3 months follow-up after wound healing, including skin color, tissue depression, and hair growth.

### 2.5. Statistical analysis

SPSS 22.0 statistical software was used for statistical analysis. Data are presented as mean and standard deviation for quantitative variables, and qualitative variables are presented as frequency and percentage. The means of the 2 groups were compared using the independent sample *t* test. In contrast, the paired sample *t* test was performed to compare the quantitative variables between the 2 dependent groups (before and after treatment). Finally, Fisher exact test was used to compare the distribution of qualitative variables among the various groups. *P* < .05 was considered statistically significant.

## 3. Results and discussion

### 3.1. Patient demographics and scalp defects characteristics

Overall, 24 patients (range, 24–95 years) with scalp defects and skull exposure were equally divided into PRG and TET groups. The main causes of scalp defects combined with skull exposure were trauma, pressure ulcers, and poorly healed incisions. The scalp defect sites were the frontal, parietal, occipital, and temporal regions. Patient demographic and scalp defect characteristics are summarized in Table 1.

**Table 1:** Patient demographics and scalp defects characteristics of the PRG and TET groups.

Characteristics	PRG group (n = 12)	TET group (n = 12)
Gender (male/female)	10/2	5/7
Age (mean ± SD), yr	62.83 ± 11.92	54.42 ± 18.54
Causes of scalp defects		
Trauma	8	3
Pressure ulcer	2	6
Poorly healed incisions	2	3
Site of scalp defects		
Frontal site	3	1
Parietal site	5	1
Parietooccipital site	2	6
Temporal site	2	4
Area of scalp defect		
Small defects (<5 cm^2^)	0	0
Moderate defects (5–20 cm^2^)	9	8
Large defects (>20 cm^2^)	3	4
Average area (cm^2^)	17.50 ± 4.99	24.44 ± 14.83
Nutritional status		
TP (g/L)	61.8 ± 5.9	60.4 ± 6.5
Alb (g/L)	37.6 ± 4.2	36.8 ± 5.6
HGB (g/L)	108.8 ± 6.4	111.0 ± 5.7
Smoking history	5	3
Diabetes	6	5

Alb = albumin, HGB = hemoglobin, SD = standard deviation, TP = total protein.

### 3.2. Outcome of treatment

**Figure 3.**
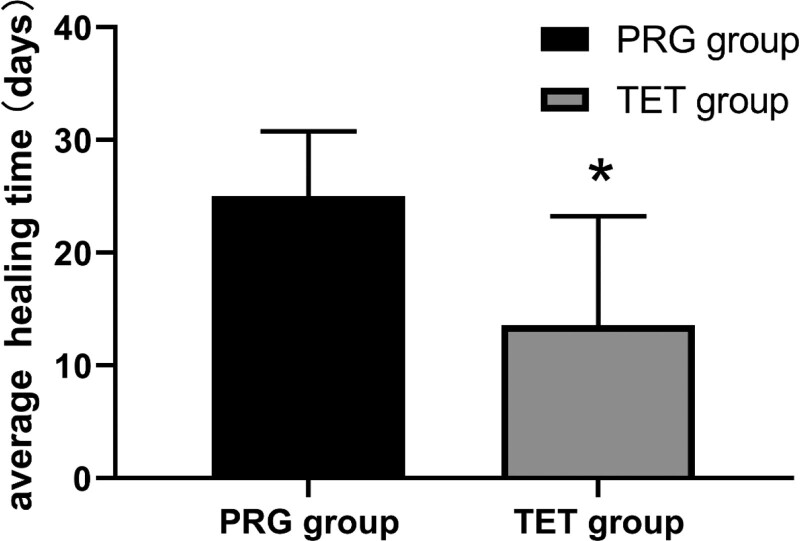
The average healing time of the 2 groups.

**Figure 4.**
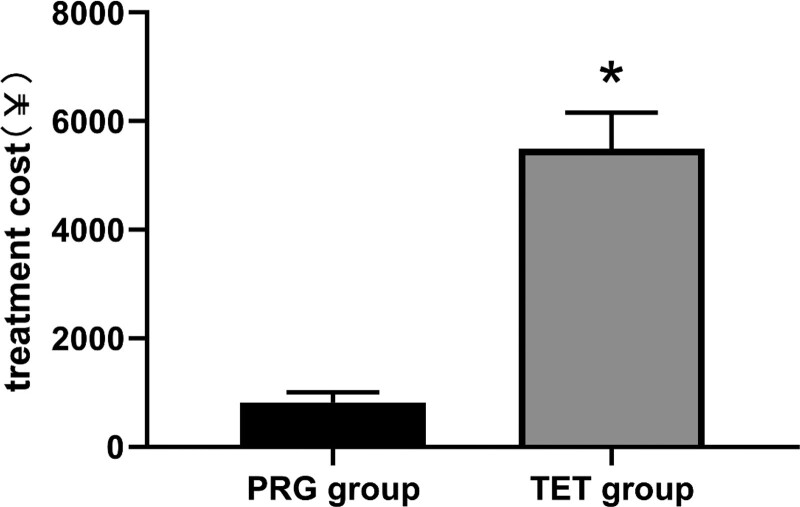
The average treatment cost in both groups.

The mean wound healing time was 25.00 ± 5.77 days in the PRG group and 13.58 ± 9.68 days in the TET group, and the difference was statistically significant (*P* < .05, Fig. 3). This indicates that the treatment period of TET was significantly shorter than that of PRG. In addition, the cost of PRG treatment was significantly lower than that of TET treatment (*P* < .05, Fig. 4), indicating that TET treatment significantly increased patients’ treatment costs.

**Figure 5.**
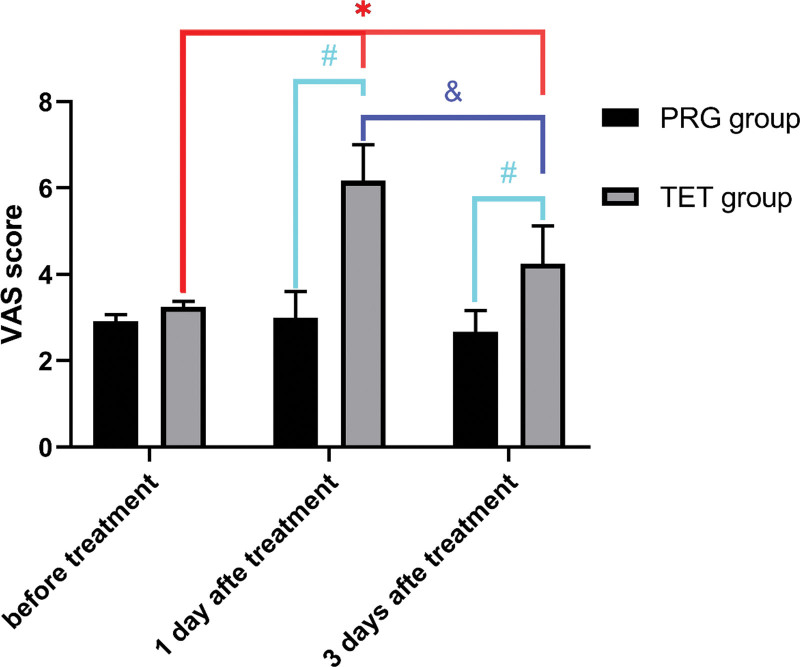
The average VAS scores of the 2 groups before and after treatment. Note: * indicates significant differences before and after treatment in the TET group (*P* < .05); # indicates significant differences compared with the PRG group 1 and 3 days after treatment (*P* < .05); & indicates significant differences between 1 and 3 days after treatment in the TET group (*P* < .05). PRG = platelet-rich gel, TET = tissue expansion technique, VAS = visual analog scale.

Pain intensity was assessed using the VAS before treatment and 6 hours and 3 days after surgery and PRG treatment. There were no significant differences in the VAS scores between the 2 groups before treatment, indicating that the wound pain intensity of the 2 groups was similar. There was no significant difference in pain intensity between the PRG group before and after treatment, indicating that PRG treatment did not increase pain. At 6 hours and 3 days after surgery, the pain intensity was significantly higher than that present preoperatively (*P* < .05, Fig. 5), indicating that TET treatment can significantly increase pain. In addition, at 3 days postoperatively, the pain intensity in the TET group was lower than that at 6 hours, indicating that postoperative pain gradually decreased over time (*P* < .05, Fig. 5).

Three months after wound healing, no obvious tissue depression was found in either group of patients, and the color of wound healing was not significantly different from that of the surrounding skin. In addition, there was no obvious hair growth in the PRG group, while hair grew normally in the TET group, except for 5 patients (*P* < .05, Table 2).

**Table 2:** Hair growth after scalp wound healing.

Groups	Hair growth	No hair growth	Total	Effective rate (%)	*P* value
PRG group	0	9	9	0	.005
TET group	7	5	12	58.33	

PRG = platelet-rich gel, TET = tissue expansion technique.

In contrast to wound repair in other parts, reconstruction of scalp defects should consider the problem of wound tissue coverage and aesthetics after surgery. Reconstruction of scalp defects is a complicated and challenging procedure for reconstructive surgeons because of limited movement of the scalp and hypovascularity of the calvaria. The correct choice of reconstruction method is often influenced by the defect’s size, depth, and location; periosteal injury; quality of the surrounding scalp tissue; hair damage; and patient comorbidities.^[1]^ Although smaller scalp defects are sutured directly to close the wound, larger defects may require advanced reconstruction methods because of the relative inelasticity of the surrounding tissue. Various methods of scalp defect reconstruction have been documented in the literature, ranging from secondary intention to free flap reconstruction, according to the area of scalp defects.^[14]^ However, traditional surgical treatment methods are complex, high-risk, and require additional skin donor areas. In addition, a large number of scars are often left in the healing site, the healing tissue is not wear-resistant, and sensory function is often difficult to restore.^[15]^ Therefore, applying TET provides a relatively simple and suitable method for the primary closure of various wounds.

A total of 22 different kinds of external tissue expansion devices have been described in the literature, which can be classified into continuous and instant external tissue expanders according to the expansion method.^[16]^ In this study, we chose 1 type of instant external tissue expander. Among the 12 patients treated with external TET in this study, 10 were sutured directly after skin stretching, and 2 healed with routine dressing changes after first-stage skin stretching to reduce the wound area. The TET achieved good clinical efficacy in this study, and the main reason was that the average distance between the scalp defect wound skin margin was only 3.2 cm (range 2.3–4.4 cm). However, during the operation, it was found that once the distance between the skin edge of the wound exceeded 4 cm, the scalp defect wound could not be closed directly because of the poor mobility of the scalp, even if the stretch force was repeatedly applied. Therefore, the intermittent TET used in this study is only suitable for scalp defect wounds where the distance between the skin margins is not >4 cm.

In contrast, wounds with large defect areas can be closed using continuous TET by slow stretching for a long time, as reported in the literature.^[17]^ Additionally, the scalp defect site affects wound healing. When using TET to treat scalp defect wounds, regions with more scalp activity (i.e., temporal and suboccipital) were reported to be more prone to closure than areas with less activity (i.e., parietal and frontal).^[17]^

Generally, moderate (5–20 cm^2^) to large (>20 cm^2^) scalp defects cannot be closed by direct suture and often require surgical reconstruction,^[2]^ which is associated with risks of anesthesia and surgery and is difficult to apply to patients with poor cardiopulmonary function who cannot tolerate anesthesia. Therefore, nonsurgical treatment is the only treatment of choice for these patients. In clinical practice, most PRGs are a type of PRP gel, which are prepared by adding a coagulant in a sterile centrifuge tube containing PRP at a ratio of 1:10. As a new and important nonsurgical treatment of wounds, it has been widely used in various wound repairs.^[18]^ Unlike the preparation of platelet-rich fibrin and PRP gel, the PRG used in this study can be prepared only by a single centrifugation for 5 minutes because of the use of centrifuge tubes containing coagulant, which not only shortened the preparation time and improved clinical efficiency but also reduced the risk of infection. Gökkaya et al^[19]^ reported for the first time a case report in which trephine combined with PRP successfully reconstructed a large area of scalp defect with an exposed skull. In this study, 12 patients received PRG treatment and were finally all healed, with an average healing time of 25.00 ± 5.77 days. No obvious tissue depression was found in any patient, and the color of wound healing was not significantly different from that of the surrounding skin at the 3-month follow-up. The only drawback is that there is no significant hair growth after wound healing.

Compared with TET for the treatment of scalp defects, PRG is a nonsurgical treatment, avoiding the risks of surgery and anesthesia, having a painless treatment process, and having a low treatment cost. In addition, even if the skin edge defect exceeds 4 cm, PRG can still be administered because the distance between the skin edge and the scalp defect does not limit the treatment of PRG. More importantly, even for patients with poor general conditions such as anemia, thrombocytopenia, and hypoproteinemia, PRG treatment can still be used for wound treatment because a large number of studies have confirmed that various allogeneic platelet concentrate products are safe and effective for wound treatment in such individuals.^[20]^ The advantage of TET treatment is that the treatment time is relatively short, and hair growth is not affected after the wound is healed. We observed that the main complication of TET was postoperative pain, and most patients required postoperative analgesic treatment. Although it takes a long time for PRG to repair wounds, the treatment can be performed in an outpatient setting, compensating for the long treatment time.

However, this study had several limitations. The main limitation was that the number of patients included in this study was small. Another limitation of this study was that the scalp defect sites were not concentrated, which affected the results to a certain extent. Finally, because the wounds involved in this study were scalp defects combined with skull exposure, this study only counted the area of scalp defects and did not include the area of skull exposure.

In conclusion, compared with the treatment of scalp defects with TET, PRG treatment is not only simple and painless but also has a low treatment cost and, more importantly, it can be performed without the risk of surgery and anesthesia. However, using TET to treat scalp defects requires a careful selection of appropriate cases, especially intermittent TET.

## Acknowledgments

All authors thank all patients at Chongqing Emergency Medical Center for their participation in this study. Thanks to Dr Li Hui for his help in the statistical analysis of the data.

## Author contributions

**Data curation:** Yanan Xu.

**Formal analysis:** Yanan Xu.

**Methodology:** Jianbai Wang.

**Project administration:** Tao Ai, Yanan Xu.

**Supervision:** Jianbai Wang.

**Writing – original draft:** Tao Ai.

**Writing—review & editing:** Yanan Xu.

## References

[1] Cen HH Jin RH Yu MR. Clinical decision model for the reconstruction of 175 cases of scalp avulsion/defect. Am J Otolaryngol. 2021;42:102752. 33125900 10.1016/j.amjoto.2020.102752

[2] Cherubino M Taibi D Scamoni S. A new algorithm for the surgical management of defects of the scalp. ISRN Plast Surg. 2013;2013:1–5.

[3] Innocenti A Menichini G Innocenti M. Major scalp defect reconstruction with free flap: analysis of the results. Acta Biomed. 2022;92:e2021301. 35075095 10.23750/abm.v92i6.10089 PMC8823577

[4] Chen FF Ju HB Huang AF. Treatment of large and complicated scalp defects with free flap transfer. Biomed Res Int. 2020;2020:1–6. 10.1155/2020/2748219 PMC7199551 32382540

[5] Lembo F Cecchino LR Parisi D. Utility of a new artificial dermis as a successful tool in face and scalp reconstruction for skin cancer: analysis of the efficacy, safety, and aesthetic outcomes. Dermatol Res Pract. 2020;2020:4874035. 32765599 10.1155/2020/4874035 PMC7388001

[6] Zhang QT Xu L Liu Y. Application of skin-stretching device for closing scalp defect. J Craniofac Surg. 2022;34:374–80. 36214652 10.1097/SCS.0000000000008856

[7] Ding JK Chu FF Tang YK. Reconstruction of facial defects using a pre-expanded scalp flap: a description of the method used and outcomes of 43 patients. Front Surg. 2022;9:962737. 36003283 10.3389/fsurg.2022.962737 PMC9393413

[8] Elsaid A El-Said M Emile S. Randomized controlled trial on autologous platelet-rich plasma versus saline dressing in treatment of non-healing diabetic foot ulcers. World J Surg. 2020;44:1294–301. 31811339 10.1007/s00268-019-05316-0

[9] Uçar O Çelik S. Comparison of platelet-rich plasma gel in the care of the pressure ulcers with the dressing with serum physiology in terms of healing process and dressing costs. Int Wound J. 2020;17:831–41. 32212258 10.1111/iwj.13344 PMC7948873

[10] Smith OJ Wicaksana A Davidson D. An evaluation of the bacteriostatic effect of platelet-rich plasma. Int Wound J. 2021;18:448–56. 33476481 10.1111/iwj.13545 PMC8273594

[11] Wang GF Zhang XL Zhang ZF. Clinical study on a skin stretching technique with adjustable external fixators to treat skin defects. Medicine (Baltim). 2020;99:e22144. 10.1097/MD.0000000000022144 PMC7489720 32925769

[12] Wang X Zhang YX Kiu-Huen Ng S. Using modified skin-stretching technique as an alternative solution for the closure of moderate and extensive skin defects. Rejuvenation Res. 2021;24:407–16. 34714135 10.1089/rej.2020.2389

[13] Cheng LF Lee JT Hsu H. Simple skin-stretching device in assisted tension-free wound closure. Ann Plast Surg. 2017;78(3 Suppl 2):S52–7. 28195891 10.1097/SAP.0000000000001006 PMC5357159

[14] Yoon J Puthumana JS Nam AJ. Management of scalp injuries. Oral Maxillofac Surg Clin North Am. 2021;33:407–16. 34092461 10.1016/j.coms.2021.05.001

[15] Cui YT Yuan BM Zhang Y. Reverse-traction skin-stretching device for primary closure of large skin defects. Arch Dermatol Res. 2023;315:751–60. 36269396 10.1007/s00403-022-02408-1

[16] Tong XR Lu JY Zhang W. Efficacy and safety of external tissue expansion technique in the treatment of soft tissue defects: a systematic review and meta-analysis of outcomes and complication rates. Burns Trauma. 2022;10:tkac045. 36518877 10.1093/burnst/tkac045 PMC9741868

[17] Ramesh S Ajik S. Scalp wound closure with K wires: an alternative easier method to scalp wound closure. Med J Malaysia. 2012;67:629–30. 23770963

[18] Wang SC Li YB Li SD. Platelet-rich plasma loaded with antibiotics as an affiliated treatment for infected bone defect by combining wound healing property and antibacterial activity. Platelets. 2021;32:479–91. 32396493 10.1080/09537104.2020.1759792

[19] Gökkaya A Gorgu M. Treatment of scalp defects with a combination of trephination and platelet-rich plasma. J Tissue Viability. 2020;29:211–5. 32417023 10.1016/j.jtv.2020.04.004

[20] Liao X Liang JX Li SH. Allogeneic platelet-rich plasma therapy as an effective and safe adjuvant method for chronic wounds. J Surg Res. 2020;246:284–91. 31622885 10.1016/j.jss.2019.09.019

